# Design of electron-donating group substituted 2-PAM analogs as antidotes for organophosphate insecticide poisoning†

**DOI:** 10.1039/d3ra03087c

**Published:** 2023-11-02

**Authors:** Nalinee Kongkaew, Kowit Hengphasatporn, Yuwanda Injongkol, Pitchayathida Mee-udorn, Liyi Shi, Panupong Mahalapbutr, Phornphimon Maitarad, Ryuhei Harada, Yasuteru Shigeta, Thanyada Rungrotmongkol, Alisa S. Vangnai

**Affiliations:** a Program in Bioinformatics and Computational Biology, Graduate School, Chulalongkorn University Bangkok 10330 Thailand t.rungrotmongkol@gmail.com; b Center for Computational Sciences, University of Tsukuba 1-1-1 Tennodai Tsukuba Ibaraki 305-8577 Japan; c National Center for Genetic Engineering and Biotechnology 113 Thailand Science Park Pathumthani 12120 Thailand; d Research Center of Nano Science and Technology, Department of Chemistry, College of Science, Shanghai University Shanghai 200444 China pmaitarad@shu.edu.cn; e Department of Chemistry, College of Sciences, State Key Laboratory of Advanced Special Steel, Research Center of Nano Science and Technology, School of Materials Science and Engineering, Shanghai University Shanghai 200444 China; f Department of Biochemistry, Center for Translational Medicine, Faculty of Medicine, Khon Kaen University Khon Kaen 40002 Thailand; g Center of Excellence in Biocatalyst and Sustainable Biotechnology, Department of Biochemistry, Faculty of Science, Chulalongkorn University Bangkok 10330 Thailand Alisa.V@chula.ac.th

## Abstract

The use of organophosphate (OPs) pesticides is widespread in agriculture and horticulture, but these chemicals can be lethal to humans, causing fatalities and deaths each year. The inhibition of acetylcholinesterase (AChE) by OPs leads to the overstimulation of cholinergic receptors, ultimately resulting in respiratory arrest, seizures, and death. Although 2-pralidoxime (2-PAM) is the FDA-approved drug for treating OP poisoning, there is difficulty in blood–brain barrier permeation. To address this issue, we designed and evaluated a series of 2-PAM analogs by substituting electron-donating groups on the *para* and/or *ortho* positions of the pyridinium core using *in silico* techniques. Our PCM-ONIOM2 (MP2/6-31G*:PM7//B3LYP/6-31G*:UFF) binding energy results demonstrated that 13 compounds exhibited higher binding energy than 2-PAM. The analog with phenyl and methyl groups substituted on the *para* and *ortho* positions, respectively, showed the most favorable binding characteristics, with aromatic residues in the active site (Y124, W286, F297, W338, and Y341) and the catalytic residue S203 covalently bonding with paraoxon. The results of DS-MD simulation revealed a highly favorable apical conformation of the potent analog, which has the potential to enhance reactivation of AChE. Importantly, newly designed compound demonstrated appropriate drug-likeness properties and blood–brain barrier penetration. These results provide a rational guide for developing new antidotes to treat organophosphate insecticide toxicity.

## Abstract 2

Design of novel 2-pralidoxime analogs by incorporating electron-donating groups onto its pyridinium core to enhance blood–brain barrier permeability and binding susceptibility toward acetylcholinesterase with paraoxon bound.

## Graphical abstract

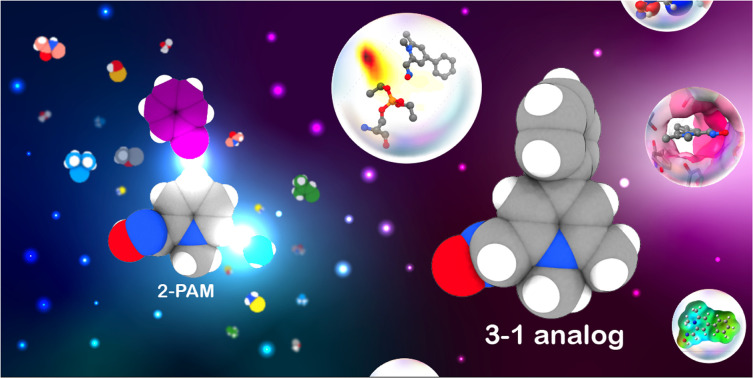

## 1 Introduction

Due to the increasing demand for food, higher crop yields have been produced, leading to the prevalent use of organophosphate (OPs) pesticides in agriculture for insect pest management. The Asia-Pacific region countries that utilize the most pesticides include China, India, Vietnam, and Thailand.^1^ The World Health Organization reports that OPs are accountable for more than 200 000 fatalities and 3 million poisonings annually.^2^ OPs can infiltrate the body through ingestion, skin absorption, and inhalation and quickly move to specific toxicity target organs within the nervous system.^3^ Acetylcholinesterase (AChE) is an enzyme responsible for terminating the activity of acetylcholine (ACh).

The primary toxic effect of OPs is the inhibition of AChE by phosphorylation of the serine hydroxyl group, resulting in decreased AChE activity caused by acetylcholine discharge. The resulting pathophysiology comprises respiratory arrest, seizures, and often death due to the paralysis of the diaphragm and intercostal muscles.^4,5^

Two procedures for treating OPs poisoning exist, one before and one after exposure to OPs, which involve the use of an acetylcholine receptor antagonist, anticonvulsant, and acetylcholinesterase reactivator.^6^ However, these treatments have several limitations, and recent research suggests that they require further study.^7^ Pralidoxime, obidoxime, HI-6, trimedoxime, and methoxime are common oxime-based reactivators or nerve antidotes. Only pralidoxime also known as 2-pyridinium aldoxime or 2-PAM received FDA^8^ and became the first commercial antidote for treating organophosphorus nerve agents (OPNAs) in 1956.^9^ However, *in vivo* studies have shown that approximately 90% of the administered dose of 2-PAM reactivators remains in the blood and peripheral tissues due to their low efficiency in crossing the blood–brain barrier (BBB).^4,10^ Consequently, only 24–34% reactivation of AChE occurs in the brain. Therefore, there is a need to enhance the efficiency of antidotes in crossing the BBB and reactivating AChE.^11^ To overcome this limitation, the researchers endeavored to develop novel antidotes by methyl scanning on the pyridinium core of the 2-PAM template.^12^

Theoretical research has been utilized as an alternative to study the interactions between inhibited enzymes and reactivators to gain additional structural insights into these interactions. Our own N-layered Integrated molecular Orbital and Molecular mechanics (ONIOM) method is a hybrid approach that distinguishes itself from the “generic” two-layer QM/MM method (*i.e.*, the IMOMM and IMOMO methods), which is a “subtractive” or “extrapolative” scheme. ONIOM was developed by Morokuma *et al.*^13,14^ and has been applied in numerous studies to investigate binding energy, including the binding of HUP A and GAL to the binding pocket of AChE,^15^ analysis of the hybrid transfer mechanism for NiAc2,^16^ binding of ellagic acid to the CK2 protein.^17^ Similar to ONIOM methods, fragment molecular orbital (FMO) calculations provide insights into the electronic structure and energetics of complex molecular interactions in a large biomolecular system, reducing time-consuming computational costs, as shown in previous studies, the binding interaction of potent compounds to SARS-CoV-2 main protease.^18,19^ Previous studies have also shown that electron-donating groups (EDGs) can improve effectively cross the blood–brain barrier (BBB) in new series of activatable photoacoustic (PA) probes for Alzheimer's disease^20^ and the substitution of EDGs can increase electron density in a molecule through the adjacent carbon atoms' effect, making nucleophiles stronger. For example, installing a strong EDG on the *ortho* or *para* position of some polyhalogenated pyridines produced more negative changes in the system.^21^ Moreover, boosting the electron density within the structure of 2-PAM, oriented in an apical (in-line) conformation towards paraoxon, enhances interactions with several residues in the catalytic active site (CAS) residues of AChE. These residues, namely W86, Y119, Y124, Y133, E202, S203, W439, H447, Y449, and also glycines.^22^ The successful drug design has been reported, such as the substitution of benzothiadiazide for interaction with phospholipid,^23^ phenolic dendritic for antioxidant activity,^24^ and tyramine oxidase inhibition.^25^

**Fig. 1:**
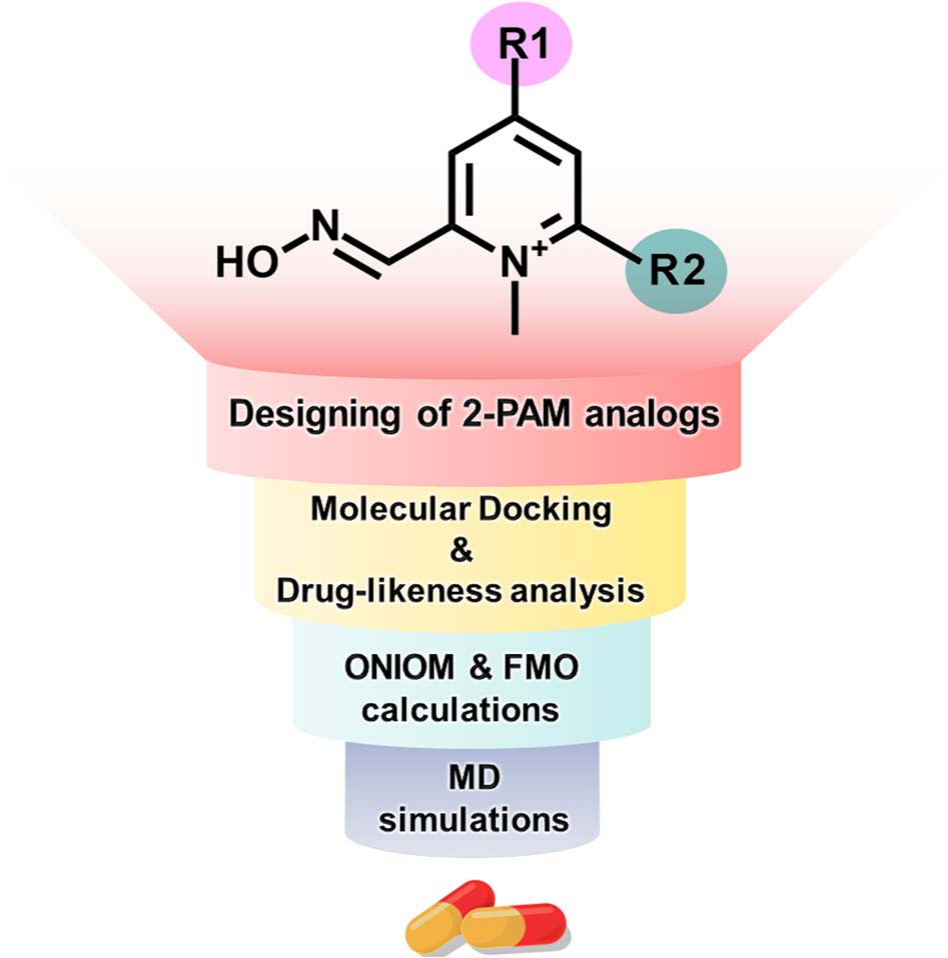
Pralidoxime antidote design and screening approach employed in this investigation.

In this study, we employed computational methods to design novel analogs of 2-PAM by incorporating EDGs onto the pyridinium core to enhance binding affinity and BBB permeability (Fig. 1). We utilized four computational techniques: molecular docking, drug-likeness analysis, ONIOM calculations, and FMO method. We evaluated the ligand-binding stability using distance-based selection molecular dynamics (DS-MD) simulations. These approaches can yield insights into the development of a more potent AChE reactivator.

## 2 Methodology

### 2.1 Designing a series of 2-PAM analogs

**Fig. 2:**
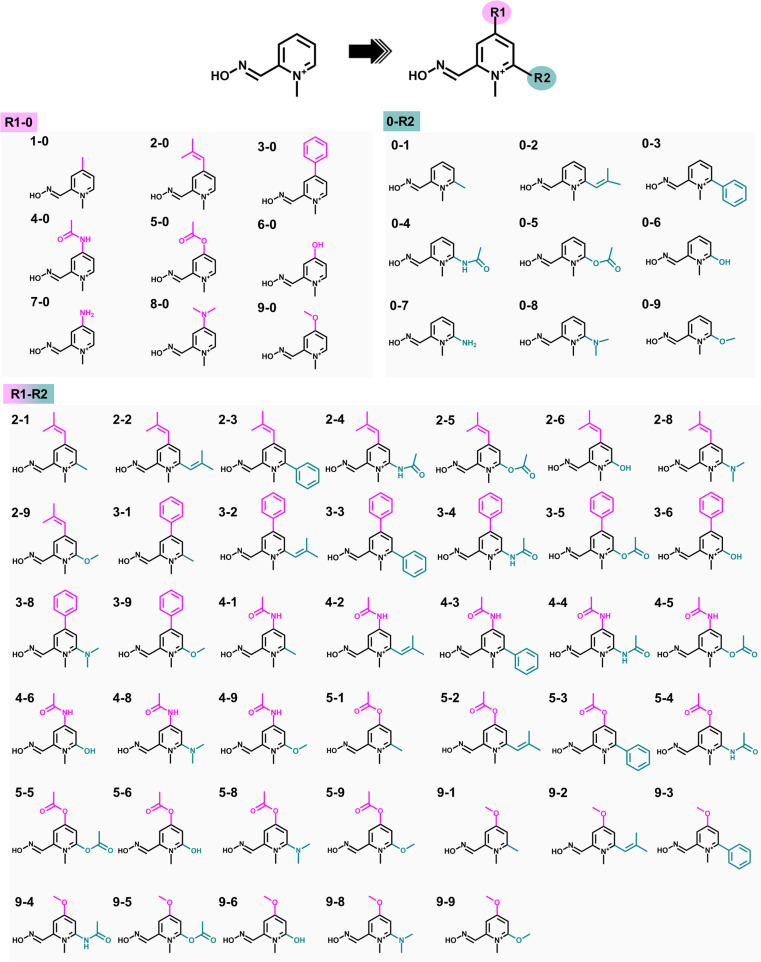
2D Chemical structures of 2-PAM analogs designed by adding nine different electron-donating groups (EDGs) at the *para* (R1) or *ortho* (R2) position on the pyridinium ring of 2-PAM. In addition, potential substituents at two positions were combined to create new analogs.

Substituting the electron-donating groups (EDGs) at the *para* (R1) and/or *ortho* (R2) positions of the pyridinium ring of 2-PAM (Fig. 2) was a strategic choice, as these positions can tolerate methylation,^12^ and the oxime binding pocket consists of high aromatic content residues.^26^ The addition of EDGs can increase electron density to the aromatic system, owing to their strong impact on the electronic structure, making it more nucleophilic, hydrophobic, and, thus, better able to cross the blood–brain barrier (BBB).^12,20^ This study included three weak, two moderate, and four strong EDGs (ESI Table S1†). The nine distinct EDGs were individually introduced at R1 or R2 positions to generate the 2-PAM analogs. Furthermore, the combination of potential substituents at both positions was considered to produce R1-R2 analogs.

### 2.2 Molecular docking and drug-likeness prediction

The study utilized the three-dimensional structure of human acetylcholinesterase in complex with paraoxon and 2-PAM in an unproductive binding mode, obtained from the Protein Data Bank with PDB entry code 5HFA.^27^ The missing amino acids at 257–264 position were added by homology modeling method using SWISS-MODEL webtool.^28^ Then, the covalent bond between paraoxon and S203 was created following the Amber Basic Tutorials – Tutorial A26.^29^ The protonation states of all ionizable residues and ligands were assigned using the PROPKA version 3.1 in PDB2PQR web server^30^ and MarvinSketch software^31^ at pH 7.0, respectively. The 3D structure of analogs was constructed using GaussView6 and subsequently optimized using density functional theory (DFT) with the B3LYP/6-31G* basis set implemented in Gaussian16.^32^ The structure of AChE with paraoxon covalently bound was prepared for refinement by undergoing relaxation through a 100 ns molecular dynamics (MD) simulation in an aqueous solution using AMBER20,^33^ following the previous study.^34^ The last snapshot, excluding waters and neutralized ions, was used for docking study.

The Autodock Vina 1.2.3 ^19,35^ was employed for molecular docking of 2-PAM and designed compounds in an “apical” orientation that fulfilled two criteria. Firstly, the distance between the oxime oxygen atom of compound and the phosphorus atom of paraoxon (*d*_OP_) was restricted to less than 8 Å. Secondly, the approaching oxime oxygen was oriented at approximately 180° to the paraoxon phosphorus and S203 hydroxyl oxygen (*θ*_OPO_).^36^ The analogs with a single substitution at the *para* or *ortho* position, denoted as R1-0 or 0-R2, were subjected to docking with 20 poses for each compound. A 15 × 15 × 15 Å cubical grid box centered on the paraoxon phosphorus was created to cover the AChE active site. The resulting pose was visualized using Accelrys Discovery Studio^37^ and UCSF Chimera,^38^ taking into account the near attack conformation (NAC) approach, where *d*_OP_ was considered.^39^

The analogs R1-0 and 0-R2, which showed better binding affinity than 2-PAM, were chosen to construct the combined analogs (R1-R2, Fig. 2). All focused analogs were then evaluated for their drug-likeness, taking into consideration various pharmacological characteristics such as molecular weight (M.W.), number of hydrogen bond donors (HBD) and acceptors (HBA), rotatable bonds (R. B.), polar surface area (PSA), and log *P*, as per Lipinski's rule of five (Ro5), as well as their ability to penetrate the blood–brain barrier (BBB)^40,41^ using freely available software such as DataWarrior^42^ and SwissADME.^43^ These characteristics are essential in the rational design of orally active drugs and central nervous system (CNS) active candidates.^44^ After that, the screened R1-R2 analogs were subjected to perform molecular docking, following the previously described protocol.

### 2.3 ONIOM and FMO calculations

Theoretical investigation of the binding energy of 2-PAM analogs bound to inhibited AChE was carried out using two-layer ONIOM calculations (ONIOM2)^13^ implemented in Gaussian16.^32^ The high-level energy of the actual system (*E*^high^_real_), which is the target energy, was approximated by eqn (1).^13^

1 *E*^high^_real_ ≈ *E*^high:low^_ONIOM2_ = *E*^high^_model_ + *E*^low^_real_ − *E*^low^_model_

**Fig. 3:**
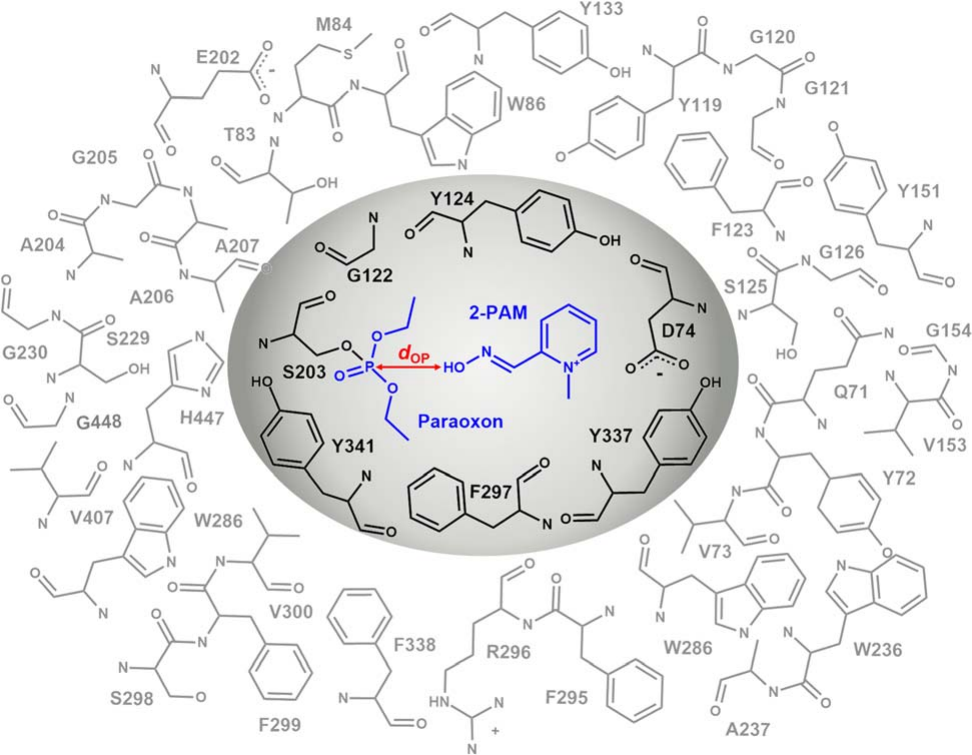
ONIOM2 scheme of the 2-PAM/inhibited AChE model system, consisting of the high-level region (gray) and low-level region (white). The *in silico* study and subsequent discussion were based on the approach where the distance *d*_OP_ between the oxygen atom of 2-PAM or its analogs and the phosphorus atom of paraoxon was less than 8 Å, and the approaching oxime group was oriented at approximately 180° to the paraoxon phosphorus and the S203 hydroxyl oxygen (*θ*_OPO_).

The inner layer, represented by the grey region in Fig. 3, comprising 2-PAM or its analogs, paraoxon covalently bonded with S203 and key interacting residues (D74, G122, Y124, F297, Y337, and Y341) suggested by iGEMDOCK,^45^ was treated at the B3LYP/6-31G* level of theory. In the outer layer, the side chains of the residues within 10 Å of the oxime oxygen, as shown in the white region of Fig. 3, were permitted to move freely, while the remaining atoms were held fixed. The outer layer, was treated with the computationally less expensive universal force field (UFF).^46^ Single-point calculations were performed using B3LYP/6-31G*:PM7 and MP2/6-31G*:PM7, taking into account the polarizable continuum model (PCM) solvation effect to obtain accurate calculations of large molecules in solution.^47^

For the computation of pair interaction energy (PIE) and its decomposition (PIEDA) between the focused compound and individual amino acids exhibiting significant binding energies, fragment molecular orbital (FMO) calculations were employed at the PCM-RIMP2/6-31G* level of theory. The PIE calculation for each compound bound to inhibited AChE was conducted utilizing the subsequent equation:

2 PIE = *E*^ES^_*ij*_ + *E*^CT+mix^_*ij*_ + *E*^DI^_*ij*_ + *E*^EX^_*ij*_ + *G*^PCM^_Sol_

Here, PIE represents the pair interaction energy of a ligand to individual residue, which can be decomposed into electrostatic interactions (*E*^ES^_*ij*_), charge-transfer (*E*^CT+mix^_*ij*_), dispersion (*E*^DI^_*ij*_), exchange (*E*^EX^_*ij*_), and solvation effect (*G*^PCM^_Sol_), called PIEDA.^48^

The molecular electrostatic potential (MEP) map of the potent analog and 2-PAM was created from the result of ligand optimization based on PCM-MP2/6-31G* level of theory, acquired through natural bond orbital (NBO) charges analysis.^49^ Additionally, the frontier molecular orbital theory of MO theory was used to determine and describe HOMO/LUMO interactions using Multiwfn.^50^

### 2.4 Molecular dynamics simulation

To assess the stability of ligand binding, we performed distance-based selection molecular dynamics (DS-MD) simulation using the concept of parallel cascade selection molecular dynamics (PaCS-MD).^51–55^ Briefly, the original PaCS-MD independently repeats multiple MD simulations from a set of initial structures, where they are selected by referring to several measures. In contrast, DS-MD repeats a single MD simulation starting from an initial structure selected by referring to the distance between a given ligand and the target protein. More specifically, DS-MD repeatedly conducts a MD simulation from the snapshot with the shortest distance for cycles, enabling one to assess the ligand-binding stability.

The optimized configuration of either 2-PAM or the most potent designed analog binding to inhibited AChE derived from ONIOM calculations was prepared using the tLeap module in AmberTools21.^56^ Gaussian16 was used to calculate the electrostatic potential charges (ESP) for the considered ligand using the B3LYP/6-31+G(d,p) method.^32,57^ The ligand charges were then fitted using restrained ESP and topology parameters with the antechamber module in AMBER20.^33^ The generalized Amber force field (GAFF)^19,58,59^ was applied to both ligands according to the standard protocol.^60,61^ The topology and coordinate files were converted to Gromacs format to conduct the DS-MD simulation.^62^ According to this technique, the distance between the *i*th and *j*th atoms (*d*_*ij*_) needs to be defined. In this case, the *d*_*ij*_ was set between the oxime oxygen of the ligand and paraoxon phosphorus (*d*_OP_), as mentioned previously. For this study, three-independent DS-MD simulations were performed. Each simulation encompassed 100 cycles, and each cycle extended for 100 ps, leading to a total simulation time of 1 ns. During the DS-MD simulations, we conducted each cycle for 100 ps, selecting the structure with the shortest value of *d*_*ij*_ as the initial structure for the subsequent cycle. The reactive trajectories of each replication were analyzed in terms of *d*_OP_ and *θ*_OPO_ using the CPPTRAJ program^63^ to assess the ligand-binding stability and mechanism.

We calculated 2D-free energy landscapes (2D FELs) of both compounds to quantitively characterize their binding processes. To calculate the 2D FELs, Markov state models (MSMs) were built using all the trajectories of each system. More specifically, 3 trials of the DS-MD simulations for each compound (100 ps × 100 cycles per the DS-MD simulation). Indeed, we used the trajectories generated after the 30th cycle (Fig. S1†) to build each MSM by specifying a lag time (25 ps) using the EMMA program.^64^ Finally, the 2D PMFs were calculated as a function of *X* and Y, which are the coordinates of *d*_OP_ and *θ*_OPO_ using matplotlib^65^ and UCSF Chimera 1.16 software.^66^

## 3 Results and discussion

### 3.1 Docking study and drug-likeness analysis of 2-PAM analogs

**Fig. 4:**
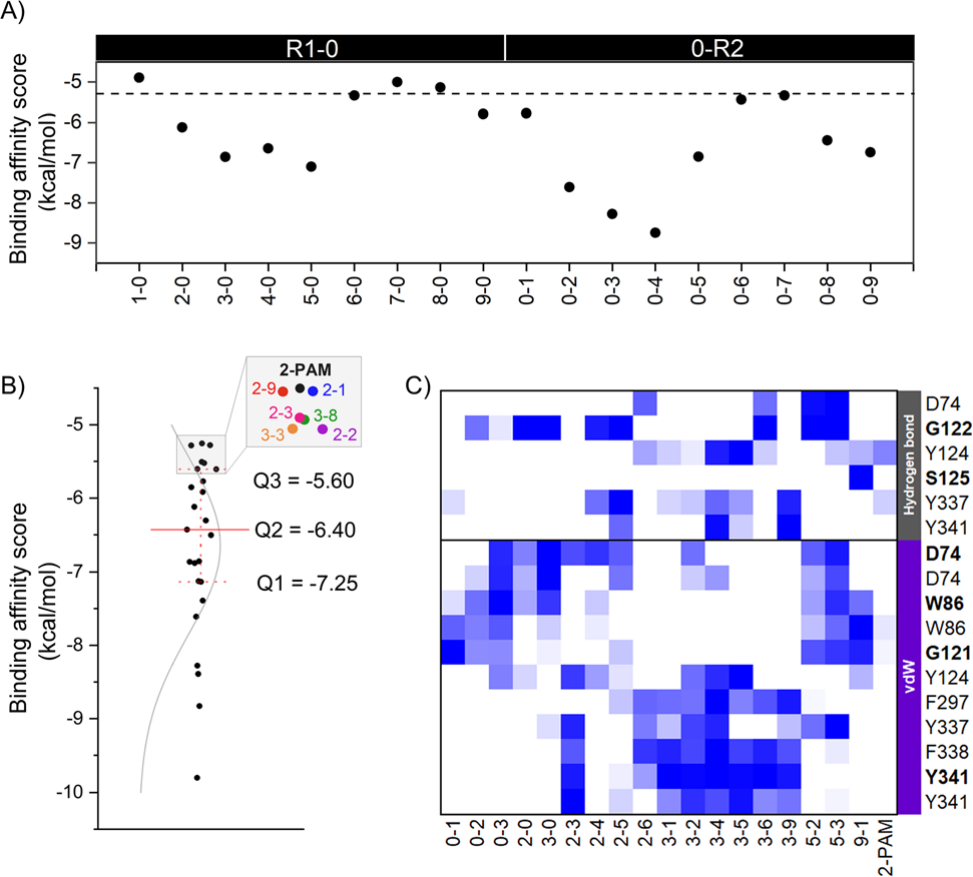
(A) The binding affinity scores of analogs containing electron-donating groups (EDGs) at either the *para* position (R1-0) or the *ortho* position (0-R2) and 2-PAM. (B) Quartile plots of binding affinity scores and density curves for 24 analogs and 2-PAM, illustrated by black dots and lines. (C) The heat map displays key interacting residues of 18 focused analogs interacting with AChE compared to 2-PAM using the iGEMDOCK program. Residues represented with bold letters indicate interactions from backbone amino acids, while residues represented with normal letters refer to interactions from side chains. Residue with a good binding affinity score is colored in descending order, blue to white.

The eighteen designed analogs with EDGs substitutions at the *para* (R1) or *ortho* (R2) position on the 2-PAM pyridinium ring in Fig. 2 were individually docked into the inhibited AChE active site using the Autodock Vina. By considering the apical conformation, the binding affinity scores for the R1-0 and 0-R2 analogs in complex with the inhibited AChE are plotted in Fig. 4A. There were five analogs from R1-0 group (2-0, 3-0, 4-0, 5-0, and 9-0) and eight analogs from 0-R2 group (0-1, 0-2, 0-3, 0-4, 0-5, 0-6, 0-8, and 0-9) that exhibited superior binding affinity scores than 2-PAM (−5.3 kcal mol^−1^, dashed line in Fig. 4A). Consequently, these potent analogs from R1-0 and 0-R2 groups were combined to form the R1-R2 analogs, consisting of 40 compounds.

A total of 53 analogs and 2-PAM were investigated for the drug-likeness and pharmacological properties using DataWarrior and SwissADME.^42,43^ The results were presented in ESI Table S2,† which included molecular weight (M.W.), number of hydrogen bond donors (HBD) and acceptors (HBA), calculated lipophilicity (*c* Log *P*), polar surface area (PSA), number of heavy atoms, and BBB permeability. Only 24 analogs, including 2 R1-0, 3 0-R2, and 19 R1-R2, passed Lipinski's rule of five criteria, which is crucial for drug discovery. These criteria included M.W. ≤ 450 Da, HBD ≤ 7, HAD ≤ 3, *c* Log *P* ≤ 5, PSA ≤ 60–70 Å, #heavy atoms 12 ≤ *X* ≤ 30, and BBB permeability.

Moreover, BBB permeability plays an important role in the treatment of 2-PAM poisoning in both the peripheral nervous system (PNS) and CNS,^36^ making it a focal point of this study. Computational calculations in Table S2† predicted a slight increase in lipophilicity (*c* Log *P*) and a slight decrease in solubility for these analogs, which is an indicator of lipophilicity.^67^ Additionally, when compared with isatin derivatives,^68^ which are novel reactivator candidates, our 2-PAM analogs demonstrated significantly higher values in terms of *c* Log *P*, especially 2-3 and 3-3. Such compounds with higher *c* Log *P* values, indicating increased lipophilicity, are more likely to cross the BBB and enter the CNS. However, this relationship is not straightforward and may be influenced by other factors such as M.W., charge, and polarity. While lipophilic compounds can cross the BBB more efficiently, they may also be more likely to accumulate in adipose tissues and have a longer duration of action. Very high *c* Log *P* values may also result in poor solubility and formulation challenges. Hence, striking the right balance between BBB permeability and physicochemical properties emerges as vital in drug discovery and development. Thus, only these 24 analogs could be promising and effective AChE reactivators by considering the permeable ability.

To further identify potent combined (R1-R2) analogs, we conducted molecular docking of 19 R1-R2 analogs towards AChE using criteria employed in the first-round screening. Analyzing the docking results statistically (Fig. 4B), we observed that both 2-PAM and 6 analogs fell within the lowest quartile (Q4) range, highlighted in the grey box. Therefore, only 18 analogs in within the Q1–Q3 range were chosen for subsequent ONIOM calculations, in comparison with 2-PAM.

In the ONIOM calculation, the important residues (D74, G122, Y124, F297, Y337, and Y341) identified through a consensus score of interacting residues ≥50% (Fig. 4C) analyzed from iGEMDOCK were set in the inner layer of the ONIOM calculations. Remarkably, we observed that G122 played a pivotal role in forming a hydrogen bond with these screened analogs.

By introducing EDGs at the *para* and/or *ortho* positions on the 2-PAM structure, the interaction profile of these analogs was confirmed to possess the capability to establish the vdW, π–π, and hydrogen bond interactions with several AChE residues in the CAS and peripheral anionic site (PAS). By considering the strength of the EDGs, the weakly donating groups (methyl (1), 2-methyl-2-butene (2), and phenyl (3) groups) showed a high contribution found in all 18 potent analogs. Particularly, the phenyl group-containing analogs served as noteworthy functional groups, facilitating interactions and improving the binding affinity score, similar to the 4-pyridine aldoxime (4-PA) in earlier research.^69^ Among the four key interacting residues identified (Y124, F297, Y337, and Y341), most analogs exhibited vdW and π–π interactions, further emphasizing the significance of these interactions in enhancing their binding capabilities due to the aromatic residues in the AChE binding pocket. There are crucial for forming a π–π interaction with the antidote and new analog, such as LLNL-02, in an apical conformation.^36^ On the other hand, single phenyl group substitution analogs (0-3 and 3-0) form the vdW to another set of residues in the binding pocket, which are D74, W86, and G121 (Fig. 4C).

### 3.2 Extrapolated QM-based binding energy and interaction

We investigated the binding energies and geometric parameters of 2-PAM analogs using B3LYP/6-31G*:UFF optimization and single point calculations with B3LYP/6-31G*:PM7//B3LYP/6-31G*:UFF and MP2/6-31G*:PM7//B3LYP/6-31G*:UFF levels of theory, with the inclusion of PCM. As discussed earlier, the reaction distance *d*_OP_ is an essential factor in determining the effectiveness of an analog as an AChE reactivator. The energetic and *d*_OP_ results obtained from the ONIOM calculations are presented in Tables 1 and S3.† The ONIOM2 (B3LYP/6-31G*:UFF) calculation reported energies ranging from −4331.56 to −4752.88 kcal mol^−1^ and *d*_OP_ ranging from 2.90 to 3.76 Å for considered analogs, while those of 2-PAM were −4292.53 kcal mol^−1^ and 4.42 Å. The PCM-ONIOM2 single-point calculations of 18 analogs compared to 2-PAM (ΔPCM-ONIOM2) revealed that the incorporation of various EDGs resulted in a substantial enhancement in binding affinity (≤−15 kcal mol^−1^) for 3 and 8 analogs in B3LYP and MP2 calculations, respectively. B3LYP includes its computational efficiency, reliability for ground state energies and geometry optimization, applicability to various systems, and ability to predict electronic excitation energies. In comparison, the MP2 method provides a more accurate description of the electrostatic, steric, polarization, and dispersion interactions between analogs and residues within the pocket.^70^ Only a 3-1 analog shows outstanding ONIOM2 results in both B3LYP and MP2 (Table 1 and ESI Fig. S2†). Hence, we selected a 3-1 analog to further insight evaluation regarding pair interaction energy (PIE) and decomposition analysis (PIEDA). The incorporation of a phenyl group onto the *para* position and a methyl group onto the *ortho* position of the pyridinium core led to a reduction in the *d*_OP_ in the 3-1 complex, with a value of 3.54 Å. The shortened *d*_OP_ could facilitate faster and more efficient reactivation *via* a nucleophilic attack on the phosphorus atom of the conjugated S203-paraoxon.^71^

**Table 1:** ONIOM2 binding energy (B. E., kcal mol^−1^) for 18 focused analogs in complex with the inhibited AChE, and the *d*_OP_ distance between the oxygen atom of 2-PAM and the phosphorus atom of paraoxon covalently bonded with the catalytic residue S203 as shown in Fig. 3. PCM-ONIOM2 results are given in ESI Table S3, while ΔPCM-ONIOM2 data are plotted in Fig. S2

	ONIOM2 B3LYP/6-31G*:UFF	ΔPCM-ONIOM2^a^		*d*_OP_ (Å)
		B3LYP/6-31G*:PM7	MP2/6-31G*:PM7	
0-1	−4331.56	−33.41	−1.77	3.39
0-2	−4448.27	22.14	12.40	3.32
0-3	−4523.29	−6.92	−19.01	3.41
2-0	−4448.28	15.54	−9.27	3.47
3-0	−4523.29	9.21	−2.66	3.76
2-3	−4681.05	−10.76	−31.65	2.90
2-4	−4658.01	2.55	−14.25	3.39
2-5	−4676.14	23.00	5.71	3.28
2-6	−4523.56	21.98	10.76	3.30
3-1	−4562.62	−32.77	−43.43	3.54
3-2	−4679.34	15.17	−19.38	3.07
3-4	−4733.04	−5.67	−24.61	2.90
3-5	−4751.17	−11.79	−28.89	3.22
3-6	−4600.48	4.33	−6.89	3.31
3-9	−4637.82	2.26	−15.87	2.95
5-2	−4676.15	25.09	11.93	3.14
5-3	−4752.88	−0.74	−19.16	3.23
9-1	−4446.09	−26.59	2.55	3.30
2-PAM	−4292.53	0	0	4.42

**Fig. 5:**
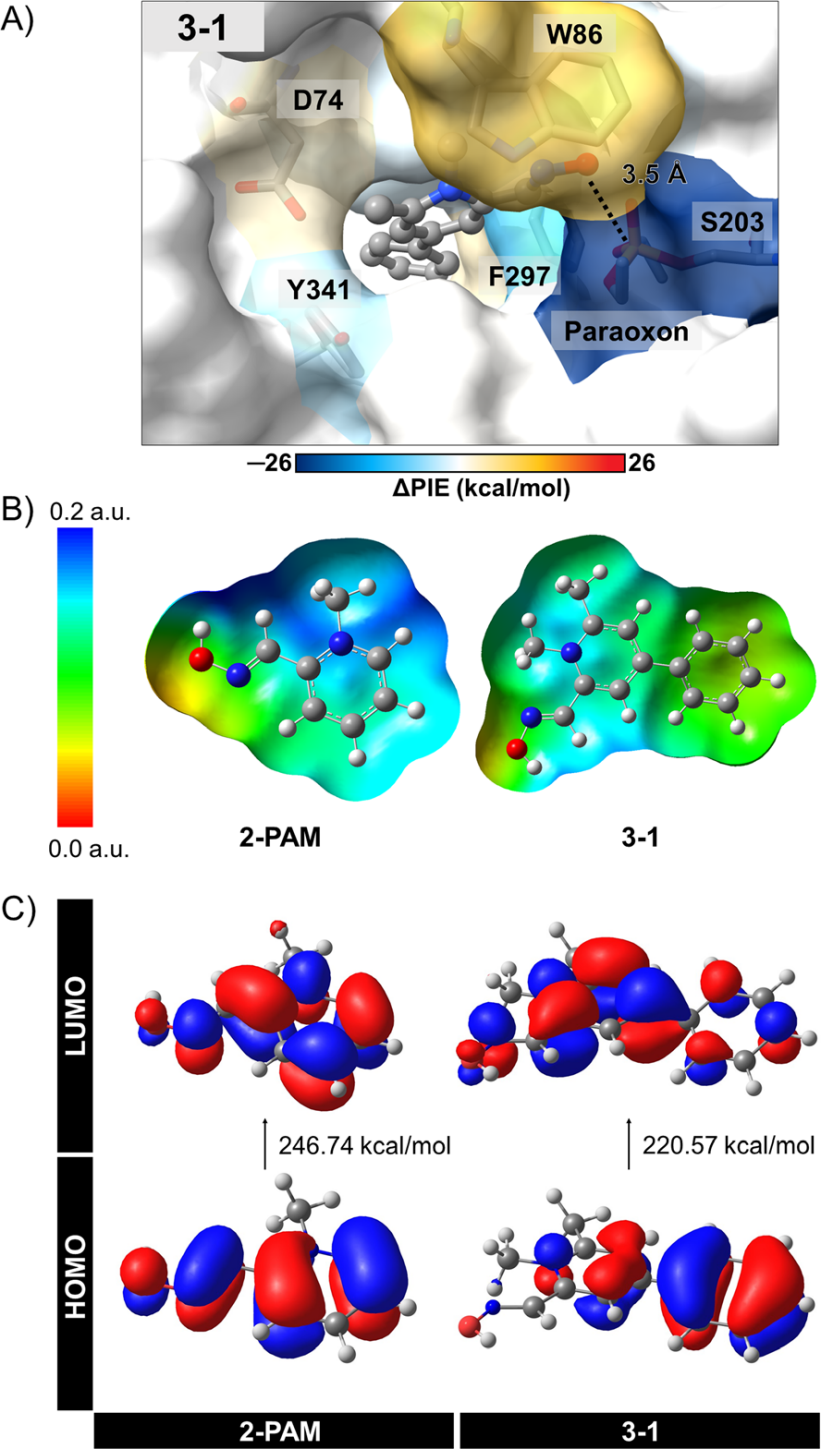
(A) Energetic fingerprints of difference (ΔPIE) in per-residue interaction energy values of potent 3-1 analogs compared to 2-PAM, as illustrated by a color gradient ranging from green to orange. (B) Molecular electrostatic potential (MEP) maps and (C) frontier molecular orbitals (HOMO–LUMO) of 2-PAM and 3-1, obtained from the single point calculation at PCM-MP2/6-31G* level of theory.

The individual interactions from PCM-RIMP2/6-31G* FMO calculations can provide insights into the specific interactions between 3-1 analog and the active site residues of inhibited AChE compared to those of 2-PAM. By analyzing these individual interactions, we can identify key interacting residues involved in binding and reactivation, thereby guiding the design of more effective AChE reactivators. We observed significant interactions of certain amino acids in the 3–1 complex (Fig. 5A, ESI Table S4, and Fig. S3†).

According to the FMO calculation, the positively charged quaternary nitrogen atom of the pyridinium ion in both compounds exhibits a robust electrostatic interaction (*E*^ES^_*ij*_) with D74 (Fig. S3A†). The pyridinium ring can form a π-donor hydrogen bond and vdW interactions with Y124, considered by dispersion (*E*^DI^_*ij*_). In the 3-1 analog, an additional phenyl ring interacts with W286 and F297 near the back door of AChE, mainly through *E*^DI^_*ij*_ and *E*^ES^_*ij*_, respectively. This interaction potentially stabilizes and maintains ligand binding after approaching the phosphorus atom of S203-paraoxon, facilitating the mechanism. Although an extra methyl group in the 3-1 analog does not frequently interact with the bound residues, it likely contributes to optimizing the ligand arrangement within the binding pocket. As a result, the oxygen atom in the 3-1 analog demonstrates a relatively strong interaction with S203-paraoxon (−33.09 kcal mol^−1^), involving electrostatic, charge exchange, dispersion, and solvation energies (Fig. S3†). These interactions could stabilize the more flexible oxime portion of the molecule.^36^

To better understand the interactions of 3-1 analog, we conducted further investigations using electronic properties, including MEP maps, NBO, and *E*_gap_ with the PCM-MP2/6-31G* computational level. The designed 3-1 involved adding more EDGs, making the –OH group more reactive and possibly resulting in more free enzymes. Fig. 5B and C illustrate the MEP maps and the molecular orbitals (HOMO and LUMO) of 3-1 compared to 2-PAM. Charge distribution in Fig. 5B showed a significant difference among these ligands, *i.e.*, adding the phenyl and methyl groups at the *para* and *ortho* positions in 3-1 led to an increased electron density distribution within the molecule. This enlargement can elevate the nucleophilic attack potential of the 3-1 analog compared to 2-PAM.

The HOMO–LUMO gap provides insights nucleophilic attack capability of these compounds (Fig. 5C). A narrower HOMO–LUMO gap indicates greater electron availability for nucleophilic interactions. In this study, 3–1 analog exhibited a HOMO–LUMO gap of 220.57 kcal mol^−1^, while 2-PAM showed 246.74 kcal mol^−1^. The *E*_gap_ of 3-1 analog showed smaller than 2-PAM, about 26 kcal mol^−1^, indicating that a small HOMO–LUMO gap can enhance charge transfer,^72^ leading to the occurrence of nucleophilic attacks and increased ability to cross the BBB. These insights could be helpful in the development of more effective AChE reactivators.

### 3.3 Molecular dynamics simulation

**Fig. 6:**
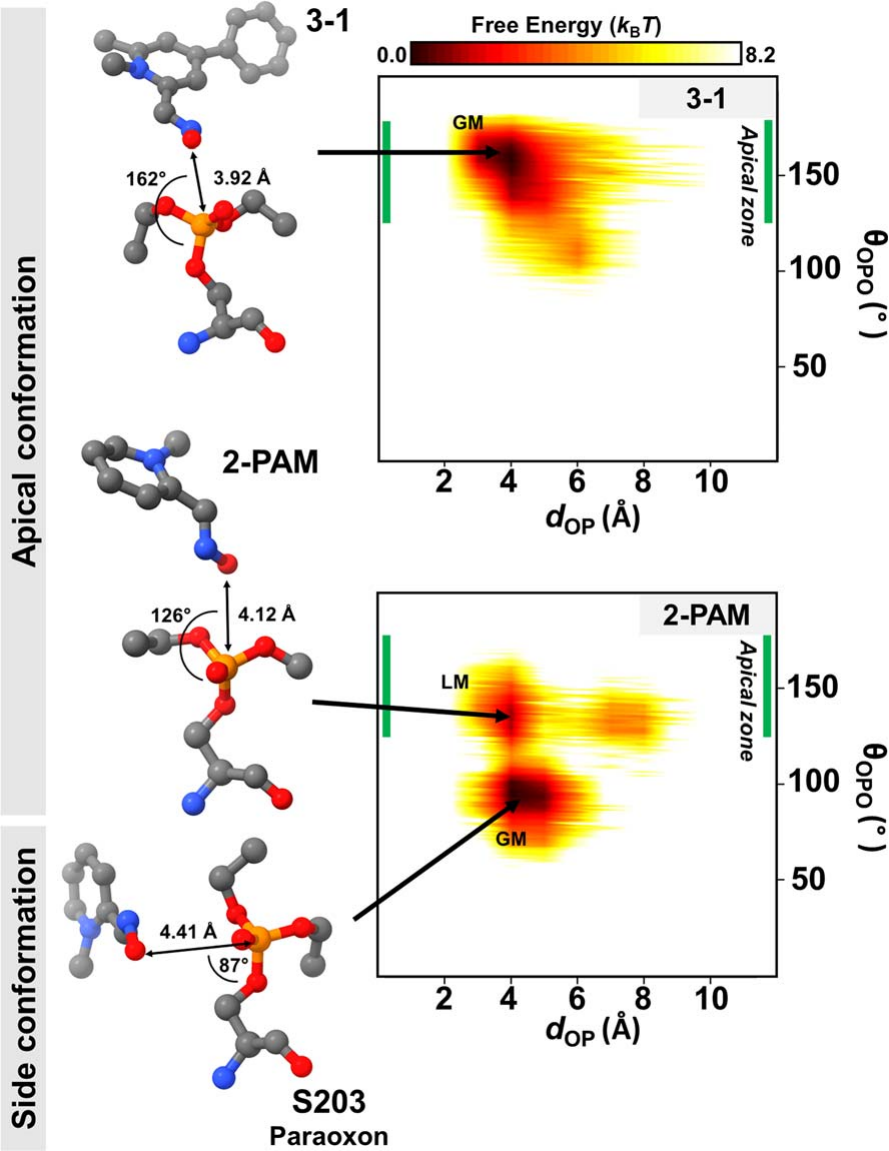
2D free energy landscape (FEL) of 2-PAM and 3-1 obtained by the DS-MD simulations plotted by *d*_OP_ and *θ*_OPO_. The apical zone (green line) in range of 120° < *θ*_OPO_ < 180° is considered by apical conformation. Representative GM and LM denote global minimum and local minimum, respectively. The value of free energy is scaled by *k*_B_*T*.

Generally, the antidote should not bind tightly to AChE because it acts as a nucleophilic agent, forming a transient covalent bond with the phosphorus of paraoxon in the active site of AChE, which is essential for the reactivation process.^73,74^ Thus, we conducted DS-MD simulations to thoroughly examine the conformational behavior and stability of the 3-1 analog binding to inhibited AChE, comparing it with 2-PAM. The conformational sampling from the DS-MD simulation was analyzed regarding free energy landscapes (FELs) as a function of *d*_OP_ and *θ*_OPO_. Notably, the optimal *θ*_OPO_ for an apical conformation is 120° < *θ*_OPO_ < 180°, as shown in the green line in Fig. 6. The 2D FELs (Fig. 6) provide valuable insights into significant ligand stability observed in 2-PAM and 3-1 analog. The initial structures of these ligands derived from the ONIOM calculation were in the apical conformation. According to the 2D FELs, only the 3-1 analog in the apical conformation (global minimum, GM) was sustained. In contrast, 2-PAM exhibited multiple states, including side (GM) and apical conformations (local minimum, LM) (Fig. 6). Once 2-PAM moves to the GM, it becomes difficult to escape from this location and return to its original orientation at LM due to a high free-energy barrier (∼5.0 *k*_B_*T*), which means 2-PAM tends to approach the paraoxon at the side conformation based on our free-energy analyses. This confirmed that the novel-designed analog, 3-1, shows a higher chance to reactivate by forming a covalent bond with the phosphorus of paraoxon, which is superior to 2-PAM. This finding firmly establishes the superiority of the novel potent analog in achieving an optimal binding orientation, especially concerning the reactivation reaction, facilitating highly efficient enzyme re-inhibition.^75^ Our comprehensive 2D FEL analyses shed light on the superior binding orientation and stability of the 3-1 analog compared to 2-PAM, offering valuable insights for designing and developing more effective AChE reactivators with enhanced enzyme re-inhibition properties.

## 4 Conclusions

This study has demonstrated the potential of electron substitution with donating groups at the *para* and *ortho* positions of the pyridinium core of 2-PAM for producing effective reactivators to treat organophosphate pesticide poisoning. Our computational methods identified the most promising functional group for these novel compounds to increase binding affinity score, AChE reactivation, and BBB permeability was the phenyl and methyl groups added to *para* and *ortho*. The key residues in the active site of inhibited AChE showed significant interactions with the 3-1 complexes were G121, Y124, W286, F297, W338, Y341, and the catalytic residue S203 covalently bonding with paraoxon calculated by the FMO method. The increase in charge distribution and nucleophilic property of 3-1 was confirmed by MEP map and *E*_gap_ analyses. The DS-MD simulations of 3-1 analog displayed high stability in the apical conformation, enhancing the reactivation mechanism. In conclusion, the superiority of the novel 3-1 analog over 2-PAM in achieving an optimal binding orientation and ligand-binding stability offers valuable insights for designing more effective AChE reactivators with enhanced enzyme re-inhibition properties, benefiting organophosphate poisoning treatments.

## Conflicts of interest

The authors report no conflict of interest, financial or otherwise.

## Supplementary Material

RA-013-D3RA03087C-s001
