# Supplementary material for: Design of electron-donating group substituted 2-PAM analogs as antidotes for organophosphate insecticide poisoning
Source: RSC Adv. 2023 Nov 2;13(46):32266–75. doi: 10.1039/d3ra03087c (PMC10620644; doi:10.1039/d3ra03087c)
Supplement: RA-013-D3RA03087C-s001 [file RA-013-D3RA03087C-s001.pdf]

Electronic Supplementary Material (ESI) for RSC Advances.
This journal is © The Royal Society of Chemistry 2023

Supplementary Information

Table S1 The electron-donating groups (EDGs) were used to substitution at the pyridinium ring of 2-PAM

Groups	Name	Structure
**Weakly donating groups**	1	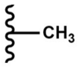
	2	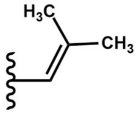
	3	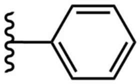
**Moderately donating groups**	4	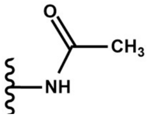
	5	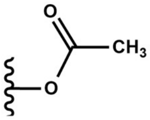
**Strongly donating groups**	6	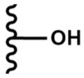
	7	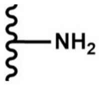
	8	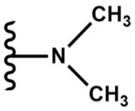
	9	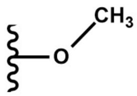

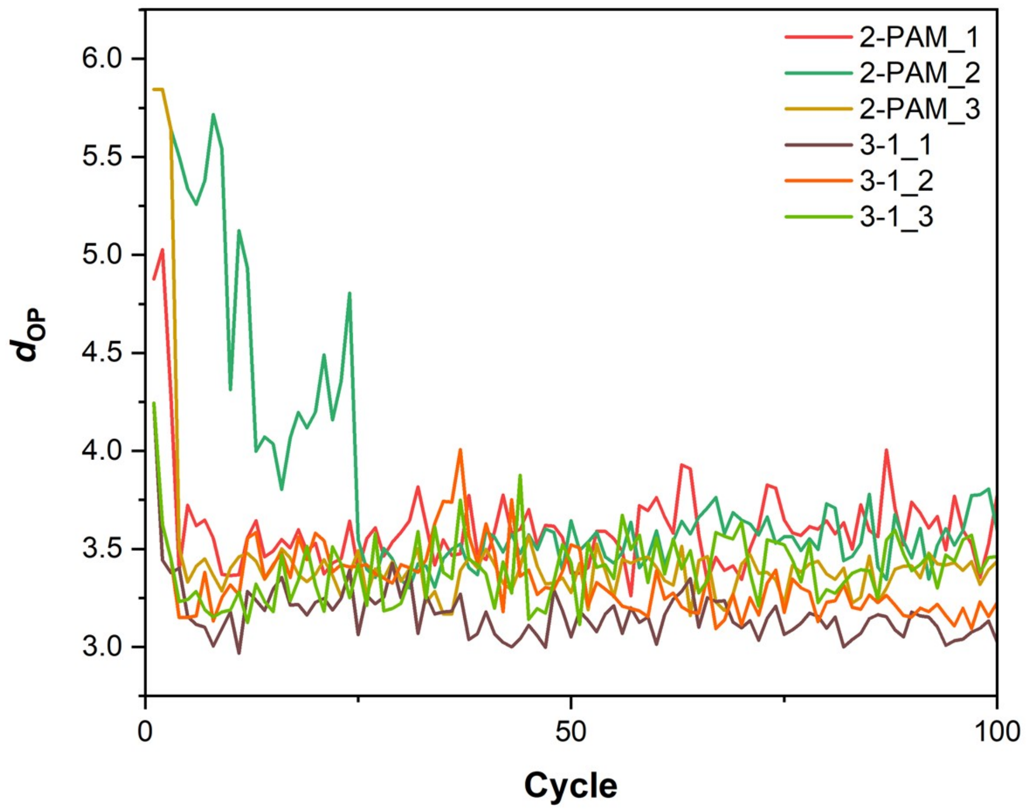

Figure S1 The d_{OP} plot of 2-PAM and 3-1 obtained from three independent DS-MD simulations

Table S2 Drug-likeness analysis for 2-PAM and designed analogs

Compound	M.W.	# HBA	# HBD	# R.B.	#heavy atom	PSA	cLogP	LLE	LELP	BBB
2-PAM	137.16	2	1	1	6	36.47	-2.66	9.52	-2.82	No
0-1	151.19	2	1	1	11	36.47	-2.26	9.08	-2.65	Yes
0-2	191.25	2	1	2	14	36.47	-0.91	7.62	-1.38	Yes
0-3	213.26	2	1	2	16	36.47	-0.91	7.58	-1.58	Yes
0-4	192.21	3	2	3	14	61.70	2.26	4.45	3.44	Yes
0-5	194.19	4	0	3	14	65.60	-2.50	9.21	-3.80	No
0-6	152.15	3	1	1	11	59.53	-2.83	9.65	-3.33	No
0-7	150.18	2	2	1	11	58.61	1.88	4.94	2.21	Yes
0-8	180.23	2	1	2	13	39.71	0.31	9.15	-3.38	No
0-9	166.18	3	0	2	12	48.53	-2.56	9.34	-3.30	No
1-0	151.19	2	1	1	11	36.47	-2.31	9.13	-2.72	Yes
2-0	191.25	2	1	2	14	36.47	-0.96	7.68	-1.46	Yes
3-0	213.26	2	1	2	12	36.47	-1.00	7.67	-1.74	Yes
4-0	194.21	3	2	3	14	65.57	-2.95	9.66	-4.49	No
5-0	194.19	4	0	3	24	65.60	-2.85	9.56	-4.34	No
6-0	151.16	3	2	1	11	52.82	2.21	4.61	2.60	Yes
7-0	150.18	2	2	1	11	58.61	1.88	4.94	2.21	Yes
8-0	180.23	2	1	2	13	39.71	-2.76	9.50	-3.88	No
9-0	166.18	3	0	2	12	48.53	-2.91	9.69	-3.75	No
2-1	205.28	2	1	2	15	36.47	-0.56	7.25	-0.92	Yes
2-2	245.34	2	1	3	18	36.47	0.79	5.82	1.57	Yes
2-3	267.35	2	1	3	20	36.47	0.79	5.78	1.75	Yes
2-4	247.29	3	1	4	18	68.40	-1.09	7.69	-2.16	Yes
2-5	248.28	4	0	4	18	65.60	-0.80	7.41	-1.60	yes
2-6	206.24	3	1	2	15	59.53	-1.14	7.82	-1.86	Yes
2-8	234.32	2	1	3	17	39.71	-0.71	7.34	-1.33	Yes
2-9	220.27	3	0	3	16	48.53	-0.86	7.52	-1.51	Yes
3-1	227.28	2	1	2	17	36.47	-0.56	7.21	-1.05	Yes
3-2	267.35	2	1	3	20	36.47	0.75	5.82	1.67	Yes

Compound	M.W.	# HBA	# HBD	# R.B.	#heavy atom	PSA	cLogP	LLE	LELP	BBB
3-3	289.35	2	1	3	22	36.47	0.75	5.79	1.85	Yes
3-4	269.3	3	1	4	20	68.40	-1.13	7.70	-2.50	Yes
3-5	270.28	4	0	4	20	65.60	-0.84	7.41	-1.87	Yes
3-6	228.25	3	1	2	17	59.53	-1.17	7.82	-2.19	Yes
3-8	256.32	2	1	3	19	39.71	-0.75	7.34	-1.57	Yes
3-9	242.27	3	0	3	18	48.53	-0.90	7.51	-1.78	Yes
4-1	206.24	3	2	3	15	61.69	2.61	4.08	4.26	Yes
4-2	246.30	3	2	4	18	61.69	3.96	2.65	7.86	Yes
4-3	268.31	3	2	4	20	61.69	3.92	2.65	8.70	Yes
4-4	249.27	4	3	5	18	90.79	1.97	4.64	3.91	No
4-5	251.24	5	1	5	18	94.70	-2.80	9.40	-5.56	No
4-6	209.20	4	2	3	15	88.63	-3.13	9.81	-5.12	No
4-8	237.28	3	2	4	17	68.81	-2.70	9.33	-5.06	No
4-9	222.24	4	2	4	16	70.92	2.19	4.46	3.84	Yes
5-1	208.21	4	0	3	15	65.60	-2.46	9.14	-4.02	No
5-2	248.28	4	0	4	18	65.60	-1.10	7.71	-2.19	Yes
5-3	270.28	4	0	4	20	65.60	-1.10	7.67	-2.45	Yes
5-4	251.24	5	1	5	18	94.70	-2.80	9.40	-5.56	No
5-5	252.22	6	0	5	18	91.90	-2.51	9.11	-5.00	No
5-6	210.19	5	1	3	15	85.83	-2.85	9.52	-4.66	No
5-8	238.26	4	1	4	17	66.01	-2.42	9.04	-4.53	No
5-9	224.21	5	0	4	16	74.83	-2.57	9.22	-4.51	No
9-1	180.20	3	0	2	13	48.53	-2.51	9.26	-3.53	Yes
9-2	219.28	3	1	3	16	41.82	4.19	2.47	7.33	Yes
9-3	241.29	3	1	1	18	41.82	4.15	2.47	8.22	Yes
9-4	223.23	4	1	4	16	77.63	-2.85	9.51	-5.01	No
9-5	224.21	5	0	4	16	74.83	-2.57	9.22	-4.51	No
9-6	182.18	4	1	2	13	68.76	-2.90	9.64	-4.08	No
9-8	210.25	3	1	3	15	48.94	-2.48	9.16	-4.06	No
9-9	196.20	4	0	3	14	57.76	-2.63	9.34	-4.00	No

Table S3 PCM-ONIOM2 binding energy (B.E., kcal/mol) at B3LYP/6-31G*:PM7 and MP2/6-31G*:PM7 levels of theory for 2-PAM and screened analogs

Compound	B3LYP/6-31G*:PM7	MP2/6-31G*:PM7
0-1	–206.04	–186.97
0-2	–150.49	–172.80
0-3	–179.55	–204.21
2-0	–157.09	–194.47
3-0	–163.42	–187.86
2-3	–183.39	–216.85
2-4	–170.08	–199.45
2-5	–149.63	–179.49
2-6	–150.65	–174.44
3-1	–205.40	–228.63
3-2	–157.46	–204.58
3-4	–178.30	–209.81
3-5	–184.42	–214.09
3-6	–168.30	–192.09
3-9	–170.37	–201.07
5-2	–147.54	–173.27
5-3	–173.37	–204.36
9-1	–199.22	–182.65
2-PAM	–172.63	–185.20

Table S4 The pair interaction energy (PIE) of the focused compounds (2-PAM and 3-1) with key interacting residues calculated by the FMO-RIMP2/6-31G*/PCM. The difference in individual interactions between 2-PAM and 3-1 (ΔPIE) is also given.

**Key interacting residue**	**FMO-RIMP2/6-31G*/PCM (kcal/mol)**		
	**2-PAM**	**3-1**	
	PIE	PIE	ΔPIE
D74	−56.16	−53.13	3.03
T83	−1.70	−1.55	0.15
W86	−11.71	−2.39	9.32
G121	−0.12	−0.93	−0.81
Y124	−3.38	−4.62	−1.23
S203-Paraoxon	−7.67	−33.09	−25.42
W286	−2.23	−3.93	−1.70
F295	−0.63	−0.63	0.01
R296	10.73	14.13	3.40
F297	−10.32	−19.17	−8.85
Y337	11.75	12.03	0.27
W338	−5.07	−5.75	−0.68
Y341	−1.19	−4.87	−3.68
PIE^{Total}	−77.68	−103.88	−26.20

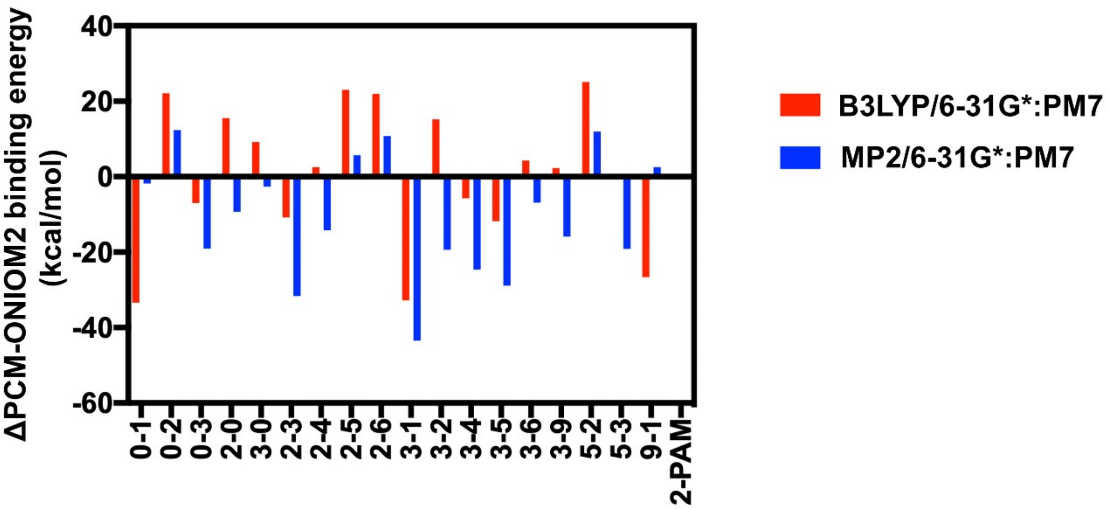

Figure S2 ΔPCM-ONIOM2 binding energy (B.E., kcal/mol) at B3LYP/6-31G*:PM7 and MP2/6-31G*:PM7 levels of theory for 18 screened analogs in complex with paraoxon-AChE relative to 2-PAM

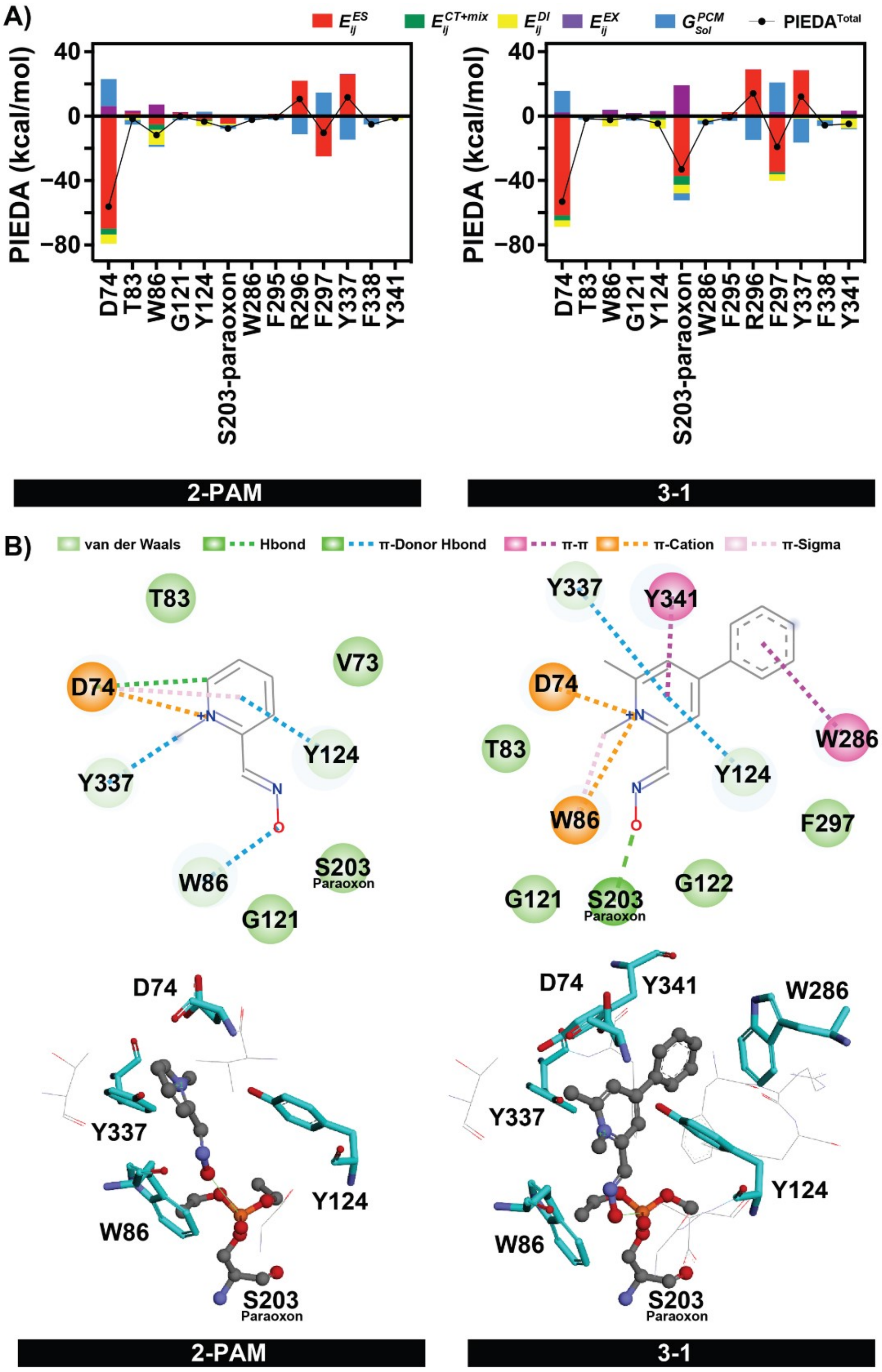

Figure S3 FMO-RIMP2/6-31G*/PCM interaction energy comparison of 2-PAM and 3-1 analog binding at the paraoxon-AChE active site. (A) Energy decomposition analysis (PIEDA) and total interaction energy (PIE^{Total}) are presented as a stacked bar graph and line plot. (B) Ligand-binding interaction in 2D and 3D perspectives are visualized using Accelrys Discovery Studio
